# Green dehydrogenation of dimethylamine borane catalyzed by cheaply copper(0) nanocatalysts without any stabilizer at nearly room temperature

**DOI:** 10.3906/kim-2101-58

**Published:** 2021-06-02

**Authors:** Ali ÖZDEMİR, Sibel DUMAN

**Affiliations:** Department of Chemistry, Bingöl University, Bingöl, Turkey

**Keywords:** Copper, dimethylamine-borane, green dehydrogenation, heterogeneous catalysis, nanocatalysts

## Abstract

In this work, the findings of the work on the green dehydrogenation behavior of molten dimethylamine borane (DMAB) catalyzed by the precatalyst copper(II) acetylacetonate (Cu(acac)_2_) in solvent-free medium (green) at near room temperature (nRT, 30.0+0.1°C) were reported. Herein, a complete study has been presented, which includes the following steps: (i) synthesis and catalytic activity of Cu(0) NCats in solvent-free medium, (ii) determination of activation energy for Cu(0) NCats catalyzed green dehydrogenation of DMAB, (iii) demonstration of catalytic lifetime of Cu(0) NCats, (iv) test of isolability and reusability of Cu(0) NCats, (v) poisoning experiments using carbon disulfide on a per-active-copper-atom basis, (vii) characterization of Cu(0) NCats by UV–vis, XRD, XPS and TEM/HRTEM/TEM-EDX spectroscopies. In addition, ATR-FTIR and ^11^B NMR techniques were use to characterize the cyclic aminoborane product obtained as a result of dehydrogenation of dimethylamine-borane.

## 1. Introduction

With the increasing demand for energy in recent years, scientists have started to show great interest in alternative energy sources. Accordingly, hydrogen stands out as a sustainable, renewable, and clean energy source [1, 2]. Hydrogen, which is a secondary energy source of the future, is considered as the most important energy carrier [3, 4, 5]. However, the most important issue for hydrogen is the storage problem, which limits its applicability. In this context, amine-boranes with high hydrogen storage capacity remain promising [6, 7, 8]. Dimethylamine borane (DMAB, (CH_3_)_2_NH•BH_3_) as solid hydrogen storage agent occupies a special position among the amine-boranes because of its gravimetric hydrogen storage capacity (6.9%) and its excellent stability and environmental friendliness [9, 10, 11]. Also, DMAB releases one equivalent dihydrogen (H_2_) using a suitable nanocatalyst (Eq. 1).


(1) $$2{\text{Me}}_{2}\text{NH.}{\text{BH}}_{3}\overset{catalyst}{\to }{\left({\text{Me}}_{2}\text{N.}{\text{BH}}_{2}\right)}_{2}+2{\text{H}}_{2}$$

There are several techniques to produce dihydrogen using DMAB, which is one of the best solid materials for hydrogen storage [12]. Usually, dihydrogen is acquired by dehydrogenation of DMAB catalyzed by convenient metal nanocatalysts at high temperatures or in a solvent environment [13, 14, 15, 16, 17, 18, 19, 20, 21, 22, 23, 24]. Previously, we reported the effect of using oleylamine on copper(0) nanoparticles for the catalytic dehydrogenation of DMAB in solvent (5.0 mL toluene) and at relatively high temperature (50.0 + 0.1°C) [25]. In this study, we provided a full transformation of DMAB (Me_2_NHBH_3_) to cyclic aminoborane ([Me_2_NBH_2_]_2_) and obtained one equivalent dihydrogen with TOF_app_ (initial apparent turnover frequency) values of 158 and 71 h^−1^ for copper(0) nanoparticles with and without oleylamine, respectively [25]. More importantly, for the dehydrogenation of DMAB in the solution medium, the Arrhenius activation energies of copper(0) nanoparticles with and without oleylamine were calculated as Ea = 19 ± 2 and 88 ± 2 kJ mol^−1^, respectively, and these values were comparable to the others (Table, see later). Although copper nanoparticles with and without oleylamine used for the dehydrogenation of DMAB have high activity at 50.0 + 0.1°C, the Cu(0) nanoparticles achieved by reduction of copper(II) acetylacetonate in this study do not require stabilizers and solvents, and the dihydrogen is released upon dehydrogenation of DMAB at a lower temperature (nRT, 30.0 ± 0.1°C). Nowadays, it is very important to perform economic studies, and, with this study, four important economic advantages were obtained: (i) no solvents, (ii) no stabilizers, (iii) no expensive metals, and (iv) no high temperatures. The results are qualitatively compared with the catalytic effects of copper catalysts reported previously (see later). However, our recent study has shown that there is efficient and effective dihydrogen release in the dehydrogenation of DMAB even at temperatures below nRT in solvent-free environments using catalysts such as nickel (25.0 + 0.1°C) [26] and ruthenium (35.0 + 0.1°C) [27]. The literature survey shows that although there are a few copper nanoparticles or complexes synthesized using amine-boranes in solvent environment or at high temperature, there are no reports of using copper metal as a catalyst for green (solvent-free) dehydrogenation of DMAB [28, 29]. As in previous studies, DMAB has two important roles as stabilizer and reducing agent in the synthesis of Cu(0) nanocatalysts (NCats) throughout the dehydrogenation reaction. Moreover, an experiment was carried out with the calculations of TOF (total frequency) and TON (turnover number) values to determine the catalytic lifetime of Cu (0) NCats. The result of this experiment showed that the Cu (0) NCats provided comparable TOFapp (47.7 h^−1^) and TON (456.5 at 50 h) values for green dehydrogenation of DMAB in nRT. Cu(0) NCats exhibited high activity and stability for the dehydrogenation reaction throughout the catalytic run thanks to the stabilizing property of molten DMAB and maintained their initial activity at 81% even after the 5th run with full conversion in the green dehydrogenation of DMAB. Cu(0) NCats and by-products of DMAB formed after the dehydrogenation reaction were characterized by ^11^B NMR, UV–vis, ATR-FTIR, XRD, XPS, and TEM/HRTEM/TEM-EDX techniques. The heterogeneity of Cu(0) NCats synthesized from the green dehydrogenation of DMAB was tested using 0.1 equiv of carbon disulfide per copper atom. In order to calculate the rate law and activation energy (E_a_), kinetic studies were performed throughout the green dehydrogenation of DMAB catalyzed by Cu(0) NCats depending on substrate amounts, catalyst amounts and temperature. Thus, it can be assumed that highly active Cu(0) NCats are a good option for the green dehydrogenation of DMAB at nRT and will make an important contribution to the literature.

## 2. Experimental method

### 2.1. Materials

Cu(acac)_2_ (Copper(II) acetylacetonate, 97%), dimethylamine-borane complex (DMAB, Me_2_NHBH_3,_ 97%), carbon disulfide (CS_2_, ≥99%), THF-d_8,_ and hexane (99%) were bought from Sigma-Aldrich. Ethanol was bought from Merck. Acetone was used to clean all glass materials and Teflon-coated magnetic stir bars. All materials were rinsed a few times with copious amounts of distilled water and then dried at high temperature (110°C) in the oven during the night.

### 2.2. Characterization

Prior to transmission electron microscopy (TEM) analysis, nanocatalysts were washed by repeated centrifugation in 10 mL ethanol and redispersed by ultrasonication in 3×15 mL hexane. TEM instrument (JEM-2010F (JEOL), 200 kV, 4.5 Å point resolution) was used to acquire TEM, HRTEM, and TEM-EDX images of these prepared nanocatalysts. By withdrawing 0.5 mL aliquots of Cu(0) NCats dispersed in hexane, they were transferred to deposit on silicon oxide coated copper grids TEM using a disposable polyethylene pipette. These grids were immersed in the solution for five seconds and then the volatiles were evaporated under an inert gas atmosphere. Magnifications of the nanocatalysts were performed between 100 and 400 k. Assuming that the nanocatalysts were spherical, the diameters of the individual particles were calculated from the corresponding surface area. Size scattering was expressed as mean diameter ± standard deviation. ^11^B NMR spectra belong to DMAB and side product of DMAB (near 0.01 mg) obtained from the reactor in THF-d_8_ were recorded using Bruker Avance DPX 400 MHz spectrometer (128.15 MHz), and boron trifluoride etherate (BF_3_(C_2_H_5_)_2_O) was used as an external reference for them. Powder X-ray diffraction (XRD) patterns were recorded 2θ in the range of 5 to 90° using the Rigaku Ultima-IV diffractometer (CuKα, λ = 1.54051 Å, 40 kV, 55 mA) at 25 °C (room temperature, RT). To describe the elemental surface composition and chemical bonding properties, the XPS spectrum was recorded with a Specs-Flex XPS photoelectron spectrometer using an Al-coated anode of toroidal quartz single crystal design. The transmission energy values can be continuously selected from a minimum of 1 eV to 400 eV. UV–visible spectra of precursor Cu(acac)_2_ and Cu(0) NCats resolved in 20 μL hexane were recorded in the spectral range of 200–800 nm using a Shimadzu 1800 double beam spectrometer. The infrared spectra of DMAB and its by-products were recorded using a Perkin Elmer A 100 ATR/FTIR spectrometer.

### 2.3. Common processes and detailed kinetic studies for the green dehydrogenation of DMAB catalyzed by Cu(0) NCats

To perform the green dehydrogenation of DMAB under nitrogen atmosphere, an experimental setup consisting of jacketed reactor (50 mL), teflon-covered mixing bar, magnetic mixer (IKA®C-Mag HS7), and fixed temperature bath (Polyscience 12107-15 water bath) was set up. All reactions for the dehydrogenation of DMAB catalyzed by Cu(0) NCats were carried out according to the standard Schlenk technique, which used fixed amounts of molten DMAB and Cu(acac)_2_ at nRT. The jacketed reactor was closed after addition of the calculated amounts of reactants, the temperature was fixed using thermostatic bath, and, at the same time, the stirrer was rotated at 1000 rpm. This procedure was repeated for all reactions. The data of pressure versus time of hydrogen release during the catalytic dehydrogenation reaction were calculated using Microsoft Office Excel 2010 and Origin2016 and then converted into the equivalent and volume (mL) of hydrogen per mole DMAB.

Kinetic studies of the dehydrogenation of DMAB catalyzed by Cu(0) NCats were performed in three groups depending on the amounts of DMAB and copper and temperature. The first group experiments were carried out as a function of catalyst amount, keeping the number of moles of DMAB fixed (2.0 mmol) and varying the number of moles of Cu(acac)_2_ between 0.1–0.3 mmol at nRT. The second group experiments were performed as a function of substrate amount, mol Cu(acac)2 was kept fixed (0.2 mmol) and mol DMAB was varied between 1.0–3.0 mmol at nRT. The third experiments were carried out as a functionof temperature, moles of Cu(acac)_2_ and DMAB were kept constant (0.2 and 2.0 mmol), respectively, and the temperature was varied between 25–45 °C. Arrhenius plot [30] and values of k_obs_ (observed rate constant) obtained from the slopes of the linear sections of the dehydrogenation curves were used to calculate the activation energy (E_a_).

### 2.4. Determination of catalytic lifetime for green dehydrogenation of DMAB catalyzed by Cu(0) NCats

The value of the total turnover number (TON) was used to determine the lifetime of Cu(0) NCats throughout green dehydrogenation of DMAB. In order to determine lifetime of Cu(0) NCats during green dehydrogenation of DMAB, a new experiment was started with 0.2 mmol Cu(acac)_2_ and 2.0 mmol DMAB at nRT. When the expected hydrogen gas release was complete, more DMAB was readded to the reaction medium, and this process continued in this manner until no further hydrogen gas release was monitored.

### 2.5. Heterogeneity test for Cu(0) NCats throughout green dehydrogenation of DMAB

Recent studies have clearly shown that CS_2_ has high poisoning ability and is used in heterogeneity tests of metal nanoparticles because CS_2_ adsorbs on the metal surface due to the reduced reaction medium [26]. Generally, a poisoning test is carried out as follows: In the dehydrogenation of 2.0 mmol molten DMAB in a solvent-free environment, 0.1 equiv. of CS_2_ per copper was added to in situ formed 0.2 mmol of Cu(0) NCats after the 50% conversion. By observing the rate of dihydrogen release after and before the addition of CS_2_ (0.1 equiv. per copper) to the dehydrogenation reaction, the effect of the poison was followed and measured. The poisoning experiments were terminated when dihydrogen release was no longer monitored.

### 2.6. Reusability, isolability, and bottlability of Cu(0) NCats in green dehydrogenation of DMAB

After the dehydrogenation of DMAB (2.0 mmol) was completed in the presence of Cu(0) NCats achieved from the reducing precursor Cu(acac)_2_ (0.2 mmol) at nRT, the reaction was stopped. The reaction flask was then sealed and separated from a graduated glass tube. Finally, after releasing the hydrogen pressure, it was transferred to a new reaction flask under N_2_ atmosphere. Using 20 mL of cold hexane, the solid mixture was precipitated under inert atmosphere, and these suspended particles were separated by filtration. Then, the solid was washed with ethanol (3x20 mL), the isolated colloid was dried under vacuum and obtained as a black powder. This black powder was reused by adding DMAB again at nRT to determine its catalytic activity throughout the green dehydrogenation (five runs in total).

## 3. Results and discussion

### 3.1. Achieving and identification of Cu(0) NCats in green dehydrogenation of DMAB

As is well known, the dehydrogenation of DMAB does not proceed without a catalyst, whether it is a solvent or a solvent-free environment. Therefore, we first tested the Cu(acac)_2_ salt, which we intended to use as a precursor catalyst, in the presence of various solvents for the dehydrogenation of DMAB at nRT. No formation of Cu(0) NCats and no manufacture of dihydrogen were observed with this precursor throughout the green dehydrogenation of DMAB in many solvents (THF, toluene, etc.) at nRT. By using cheap metals such as copper for the green dehydrogenation of DMAB, it is clear that special conditions are required for the classical synthesis of a highly stable and active nanocatalyst, such as increasing the temperature or using a suitable stabilizer. This assumption was confirmed by our recent work, in which oleylamine was used as a stabilizer, and highly active copper(0) nanoparticles were synthesized throughout the dehydrogenation of DMAB in toluene solution at a relatively higher reaction temperature (50.0 ± 0.1°C) [25]. Moreover, this study, like our previous studies [26,27], supports that the best results for the synthesis of active and stable Cu(0) NCats are obtained from the dehydrogenation of DMAB under solvent-free (green) environmental conditions. Consequently, Cu(0) NCats that are active and stable even after several weeks of storage were synthesized in situ without stabilizer and solvent by using Cu(acac)_2_ as a precursor throughout the dehydrogenation of DMAB at nRT. Moreover, these nanocatalysts and the dehydrogenation reaction products isolated after washing in ethanol were identified by ^11^B NMR, UV–vis, ATR-FTIR, XRD, XPS, and TEM/HRTEM/TEM-EDX techniques.

**Figure 1:**
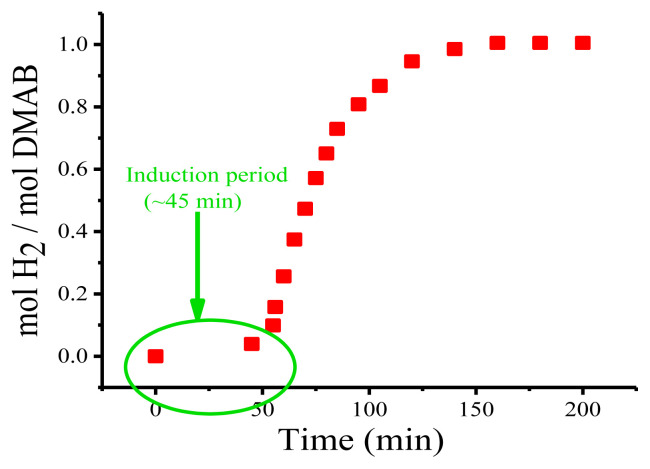
Plot of mol H_2_ per mol ofDMAB versus time for the green dehydrogenation of DMAB (2.0 mmol) starting with 0.2 mmol Cu(acac)_2_ at nRT.

The catalytic activity of Cu(0) NCats throughout the catalytic green dehydrogenation of DMAB was measured using the standard Schlenk technique as described in the Experimental Section. First, a series of experiments were performed to determine the effect of the mol ratio of DMAB:Cu(0) NCats on the green dehydrogenation of the catalytic DMAB, and it was found that this ratio is about 10 to provide and monitor the full reduction from the Cu^2+^ to the Cu^0^ oxidation state. This ratio is the best-known to obtain high activity of cheap and nonnoble metals such as copper for the catalytic green dehydrogenation of DMAB [25]. When the formation of Cu(0) NCats was completed (after ~45 min of induction period), dihydrogen release from the green dehydrogenation of DMAB started and the hydrogen pressure was manually recorded from the water level in the graduated glass tube minute by minute (Figure 1). While no dihydrogen release was monitored throughout the induction period of about 45 min required for reduction of Cu^2+^ to Cu^0^, it was clearly observed that almost all of the dihydrogen (1.0 equiv) was rapidly removed within the first 45 min after catalyst formation (hence, the total time of approximately 110 min after induction time). The plot of dihydrogen release versus time shows that the formation of Cu(0) NCats and the concomitant green dehydrogenation of DMAB occurred in a short time. Herein, it is clearly seen that Cu(0) NCats have high stability and activity in hydrogen production from green dehydrogenation of DMAB (Figure 1).

**Figure 2:**
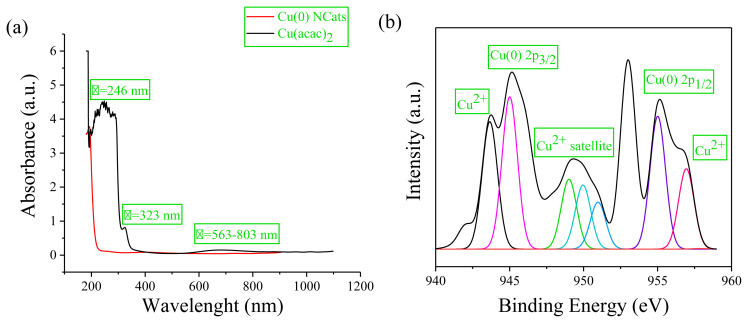
**(a)** UV-visible spectra and **(b)** high resolution XPS spectrum of 0.2 mmol Cu(acac)_2_ in hexane and after reduction by 2.0 mmol DMAB.

During the green dehydrogenation of DMAB, the formation and oxidation state of Cu(0) NCats were monitored by UV and XPS spectroscopies (Figure 2). The UV–visible spectra and color change (from blue to black) explain the rapid degradation of Cu(acac)_2_ (Cu^2+^) to copper nanocatalysts (Cu^0^) after a visible induction period of ~45 min during the dehydrogenation of DMAB. Figure 2a shows the UV-visible spectra of solutions containing Cu(acac)_2_ in hexane before and after injection into the reaction solids mixture. Examination of the UV spectra reveals three absorption peaks at λmax= 243, 323, and 563–803 nm for Cu(acac)_2_, which can be attributed to the charge transfer from metal to ligand (CTML), π-π* electron transition and d-d electron transition, respectively (Figure 2a) [31]. After the reduction of Cu^2+^ ions, these bands disappeared, and a characteristic Mie exponential decay profile for copper nanocatalysts was observed, which is consistent with the literature [32].

High-resolution XPS analysis of Cu(0) NCats (Figure 2b) confirmed the presence of copper in agreement with TEM-EDX result. Figure 2b shows that this XPS spectrum contains two prominent bands at 934.15 and 951.24 eV, assigned to Cu(0) 2p_3/2_ and Cu(0) 2p_1/2_, respectively [33, 34]. Additionally, two relatively weak peaks resulting from shaking properties were observed for Cu(0) 2p_3/2_ and Cu 2p_1/2_ around 931.76 and 953.85 eV, respectively. These peaks, attributable to Cu^2+^, are compelling evidence and indicate the presence of an 3d^9^ open shell. The observation of Cu^2+^ in the XPS spectrum can be explained as that Cu(0) NCats can be oxidized (CuO) due to exposure to air during sample preparation [35, 36]. The peak around 942.31 eV observed in Figure 2b was also attributed to the Cu^2+^satellite [37].

**Figure 3:**
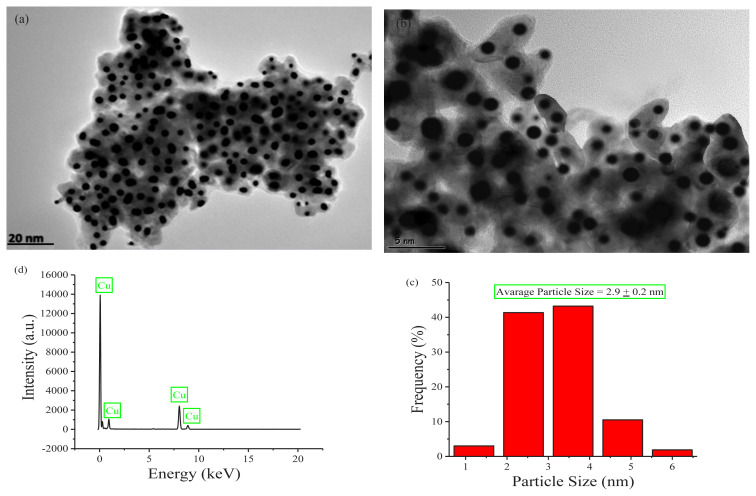
**(a)** TEM image, **(b)** particle size histogram, and **(c)** TEM-EDX of Cu(0) NCats in situ formed from the reduction of Cu(acac)_2_ during the green dehydrogenation of DMAB at nRT.

TEM images, histogram, and EDX data of Cu(0) NCats were shown in Figure 3. TEM images were used to determine the morphology and calculate the average particle size of Cu(0) NCats. TEM images recorded in different magnifications (Figures 3a, 3b) and histogram (Figure 3c) showed that 266 nontouching Cu(0) NCats in situ achieved using precursor Cu(acac)_2_ throughout the green dehydrogenation of DMAB are well distributed. The average particle size of the Cu(0) NCats obtained in situ was calculated to be 2.9 ± 0.2 nm by single counting from the TEM image (Figure 3c). The particle size of Cu(0) NCats is comparable to other catalysts, and it can be clearly seen that it is smaller than that of most other catalysts (see Table). As can be seen from the EDX data (Figure 3d), the nanocatalyst sample contains an only copper element. Thus, it can be said that our Cu(0) NCats are not oxidized during TEM sampling (see Section 2.2., The TEM grid also contains Cu) [26,27].

**Figure 4:**
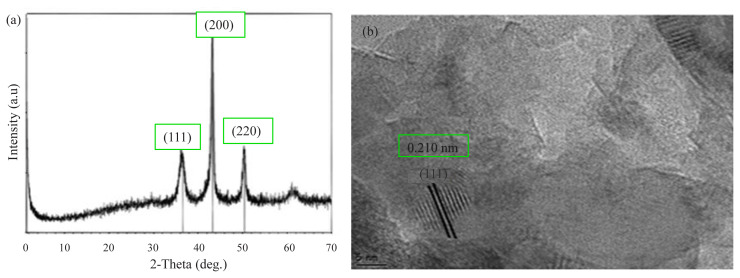
**(a)** Powder XRD pattern and **(b)** HR-TEM image of Cu(0) NCats in situ generated from the reduction of Cu(acac)_2_ during green dehydrogenation of DMAB.

Figure 4 shows the powder XRD pattern and HR-TEM analysis of Cu(0) NCats synthesized in situ during the green dehydrogenation of DMAB. In the powder XRD pattern, it was observed that three reflection peaks were centered at 2θ = ~ 36.58, 43.28, 50.42°, and these peaks were assigned to Cu (111), (200) and (220) planes, indicating a face-centered cubic structure in the crystal lattice (Figure 4a) [^38^, ^39^]. The Cu(111) diffraction peak of the obtained nanocatalyst was used to calculate the average crystallite size and lattice parameter value of the metal nanocatalysts. Also, the Scherrer formula was used to calculate the average crystallite size of Cu(0) NCats, and its size was calculated to be 2.78 nm. It can be clearly seen that this diameter is pretty close to the diameter (2.9 nm) calculated using the TEM image. HR-TEM analysis was used to investigate the crystallinity of the Cu(0) NCats synthesized in situ during the green dehydrogenation of DMAB (Figure 4b). Accordingly, no agglomeration was observed for the Cu(0) NCats that were well distributed in hexane. As can be seen from Figure 4b, the plane of Cu(111) on the prepared nanocatalysts has an interval distance of 0.210 nm and is exceedingly close to the Cu(111) interval distance of 0.209 nm, and this finding is in agreement with the literature [25, 40, 41, 42].

**Figure 5:**
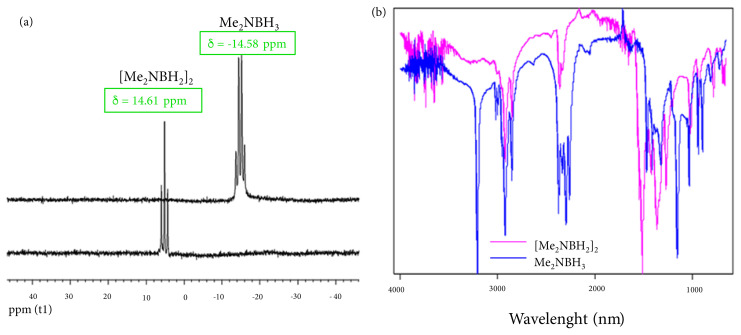
**(a)** ^11^B-{^1^H}-NMR and (b) ATR-FTIR spectra of the solid mixture containing DMAB (2.0 mmol), Cu(acac)_2_ (0.2 mmol) before and after catalytic dehydrogenation reaction.

The product obtained after catalytic dehydrogenation of DMAB was properly characterized by ^11^B-{^1^H}-NMR and ATR-FTIR spectroscopies (Figure 5). ^11^B-{^1^H}-NMR spectra were recorded after preparation as described in Section 2.2 and used to determine the product obtained after the dehydrogenation reaction of DMAB (bottom) and pure DMAB (top). As shown in Figure 5a, the resonance signal of DMAB at d = −14.58 ppm was replaced by the signal at d = 4.61 ppm of the [Me_2_NBH_2_]_2_ (cyclic dimer) after the green dehydrogenation reaction. Also, ATR-FTIR spectra (Figure 5b) were used to show differences between pure DMAB and the product formed after the dehydrogenation reaction. According to this, the typical N-H bending and N–H stretching absorption bands of pure DMAB disappeared at 1155 and 3210 cm^−1^, respectively, while its B–H stretching bands at 2377 and 2271 cm^−1^ were shifted to 1960 cm^−1^ at the end of the dehydrogenation reaction [^43^]. Furthermore, the newly formed B-N stretching band at 1615 cm^−1^ in the FTIR spectrum of the [Me_2_NBH_2_]_2_ can be explained as a change in DMAB after the dehydrogenation reaction. All these data in the ATR-FTIR and ^11^B-{^1^H}-NMR spectra show that Me_2_NHBH_3_ is fully transformed to the [Me_2_NBH_2_]_2_.

### 3.2. Calculation of activation energy from kinetic studies performed for green dehydrogenation of DMAB catalyzed by Cu(0) NCats

The kinetic study of the Cu(0) NCats formed in situ during the dehydrogenation of DMAB was carried out in three sets by observing the dihydrogen release as a function of catalyst and substrate amounts and reaction temperature.

**Figure 6:**
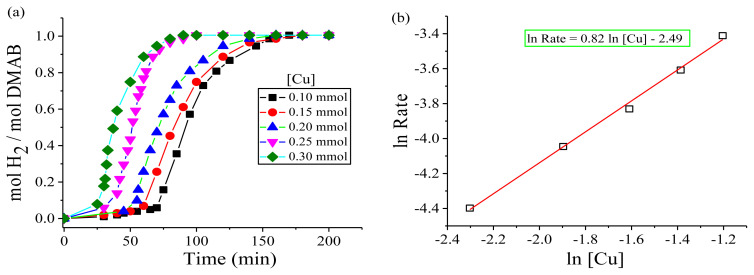
**(a)** mol H_2_/mol DMAB versus time graph depending on the various amounts of Cu(acac)_2_ for green dehydrogenation of DMAB (2.0 mmol) at nRT. **(b)** Plot of hydrogen generation rate versus amount of copper, both in logarithmic scale.

In the first set, while the reaction temperature and the amount of DMAB were fixed at nRT (30.0 ± 0.1°C) and 2.0 mmol, respectively, the effect of catalyst amount on the dihydrogen release rate in the dehydrogenation of DMAB was investigated by varying the initial amount of copper in the range of [Cu]_0_= 0.10–0.30 mmol (Figure 6). Figure 6a indicates the plots of time versus mol of hydrogen per mol of DMAB throughout dehydrogenation of DMAB, which was performed starting from a constant substrate amount of 2.0 mmol and various amounts of Cu(acac)_2_ at nRT. An induction period (~45 min) observed during the green dehydrogenation of DMAB can be explained as the time required for the formation the catalytically active copper nanocatalysts after the reduction of Cu(acac)_2_. As expected, the slope calculated from the linear part of the plots showed that there is a linear relationship between the dihydrogen release rate and the amount of Cu(acac)_2_ after the induction time. Figure 6b demonstrates the plot of the logarithmic initial rate of dihydrogen release, calculated from the slope of the plots in Figure 6a, versus the logarithmic initial amount of copper after induction period. The slope resulting from a linear fit of the plot is nearly 0.82, indicating that the dehydrogenation of DMAB is first-order with respect to the amount of Cu(acac)_2_.

**Figure 7:**
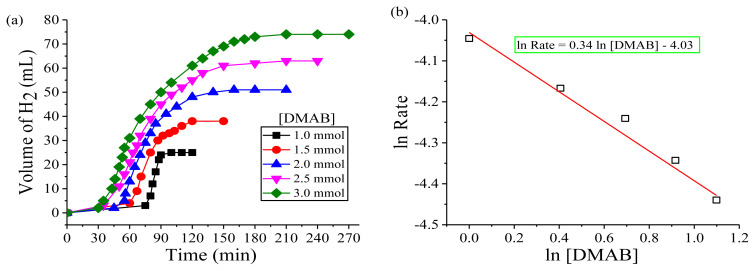
**(a)** Plots of H_2_ volume versus time for the green dehydrogenation of DMAB in various amounts in the presence of Cu(0) NCats (0.2 mmol Cu(acac)_2_) at nRT. **(b)** Plot of hydrogen generation rate versus amount of DMAB, both in logarithmic scale.

In the second set, the relationship between initial dihydrogen release rate and substrate amount was investigated by varying the initial amount of DMAB ([DMAB]_0_= 1.0–3.0 mmol) at constant copper amount (0.2 mmol) and temperature (nRT) (Figure 7). As shown in Figure 7a, the dihydrogen volume increased with the increasing amount of DMAB, but the induction period decreased throughout the dehydrogenation of DMAB. Plotting the logarithmic initial dihydrogen release rate versus the logarithmic initial DMAB amount in Figure 7b gives a straight line with a slope of about 0.34. This demonstrates that the dehydrogenation of DMAB is about half-order respect to the DMAB amount. In view of these results, the catalytic rate law for the dehydrogenation of DMAB catalyzed by Cu(0) NCats can be written as in Eq 2.


Eq 2 $$\text{Rate}={\text{k}}_{\text{app}}{\left[\text{Cu}\right]}^{0.82}{\left[\text{DMAB}\right]}^{0.34}$$

**Figure 8:**
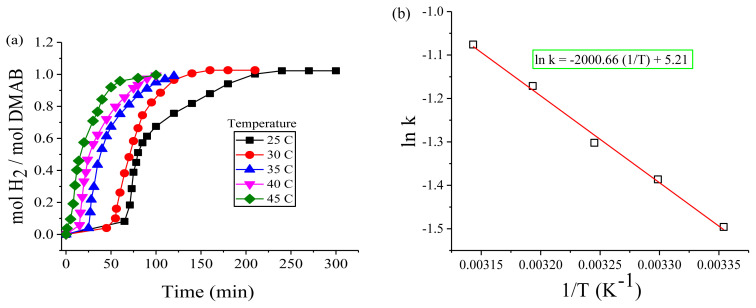
**(a)** Plots of mol H_2_/mol DMAB versus time for the green dehydrogenation of DMAB starting with 2.0 mmol DMAB plus 0.2 mmol Cu(acac)_2_ at various temperatures, **(b)** Arrhenius plot.

In the third set, the effect of reaction temperature on the initial rate of dihydrogen release was examined at different temperatures in the range of 25–45 °C, while the amounts of DMAB and Cu(acac)_2_ were kept constant at 2.0 and 0.2 mmol, respectively (Figure 8). As clearly shown in Figure 8a, the catalytic activity increased with increasing temperature, while the induction period decreased throughout the dehydrogenation of DMAB. The k_obs_ (observed rate constant) were calculated from the slopes of the dihydrogen release plots after the induction period, and these values were used to determine the activation energy for the green dehydrogenation of DMAB using the Arrhenius plot in Figure 8b. The activation energy for the catalytic dehydrogenation of DMAB catalyzed by Cu(0) NCats was calculated from the Arrhenius plot as E_a_= 16.6 ± 2 kJmol^−1^. Table demonstrates that this activation energy may be the lowest activation energy achieved in the green dehydrogenation of DMAB performed using other catalysts.

### 3.3. Lifetime study of Cu(0) NCats in the green dehydrogenation of DMAB

**Figure 9:**
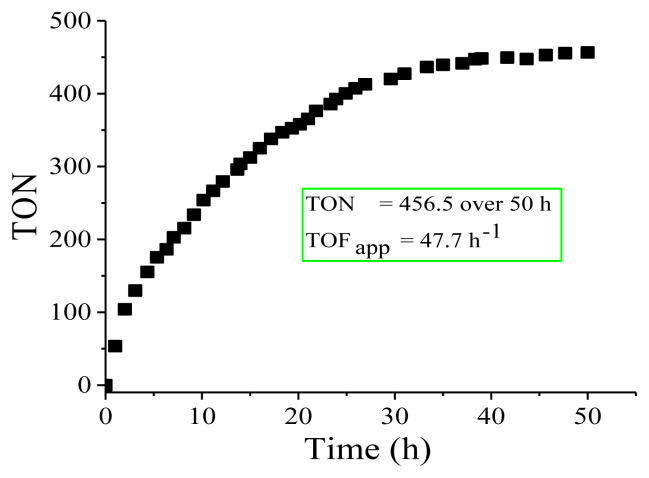
Turnover number (TON) versus time (h) plots for H_2_ generation from the catalytic green dehydrogenation of DMAB starting with 2.0 mmol DMAB plus 0.2 mmol Cu(acac)_2_ at nRT.

The lifetime study of the copper catalyst was started by adding 2.0 mmol of DMAB to Cu(0) NCats (0.2 mmol) throughout the green dehydrogenation reaction at nRT. When dihydrogen release was complete, additional DMAB was added. This process was continued until no more hydrogen was released. As expected, the induction period observed with the first DMAB addition was not observed with the other additions. As a result of this experiment, TON and TOF_app_ were found to be 456.5 over 50 h and 47.7 h^−1^, respectively during the green dehydrogenation of DMAB until deactivation of the copper catalyst because of bulk metal aggregation (Figure 9). TON and TOF values showed that Cu(0) NCats are highly stable and long-lived catalysts in the green dehydrogenation of DMAB, although no stabilizer or supporter was used. These TOF_app_ and TON values can be compared with most other catalysts synthesized by both green (solvent-free) and conventional (in solvent) methods reported hitherto (Table). As can be seen in Table, the TOF value generally increases as the particle size of the metal catalysts decreases. The reason for this can be considered that the TON and the TOF values also increase, because the small particle size of the metal catalysts has large active surface areas.

### 3.4. Heterogeneity testing for Cu(0) NCats in the green dehydrogenation of DMAB

**Figure 10:**
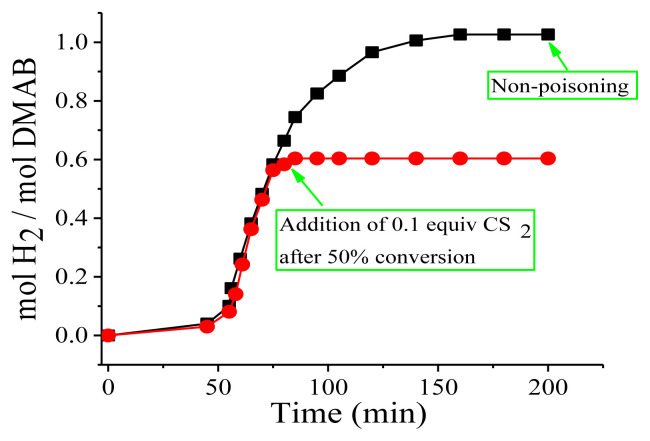
The plots of equiv dihydrogen per mol of DMAB (2.0 mmol) versus time (min) for the dehydrogenation of DMAB catalyzed by in situ generated Cu(0) NCats (0.2 mmol) with 0.1 equiv of CS_2_ poison at nRT.

To determine heterogeneity, a series of poisoning studies were performed for Cu(0) NCats in the green dehydrogenation of DMAB using CS_2_ as a poison, as detailed in Section 2.5 [44]. The use of CS_2_ is aimed to prevent the substrate from access the active site as a result of the strong binding of the poison to the metal center. Briefly, poisoning experiments for the green dehydrogenation of 2.0 mmol DMAB catalyzed by 0.2 mmol Cu(0) NCats were performed by adding 0.1 mol of CS_2_ per mol of copper under an inert atmosphere after 50% conversion of the green dehydrogenation of DMAB at nRT and then monitoring the reaction by changes in dihydrogen pressure as in previous experiments. Figure 10 reveals that Cu(0) NCats were poisoned with a strong poison such as CS_2_, and the dihydrogen release was fully halted after the addition of 0.1 equiv CS_2_ to the reaction medium. This is the clearest indication that the catalysis is heterogeneous [45]. From these results, it can be concluded that the dehydrogenation of DMAB is a heterogeneous catalysis and Cu(0) NCats are the kinetically competent catalyst.

### 3.5. Reusability and isolability of Cu(0) NCats in the green dehydrogenation of DMAB

**Figure 11:**
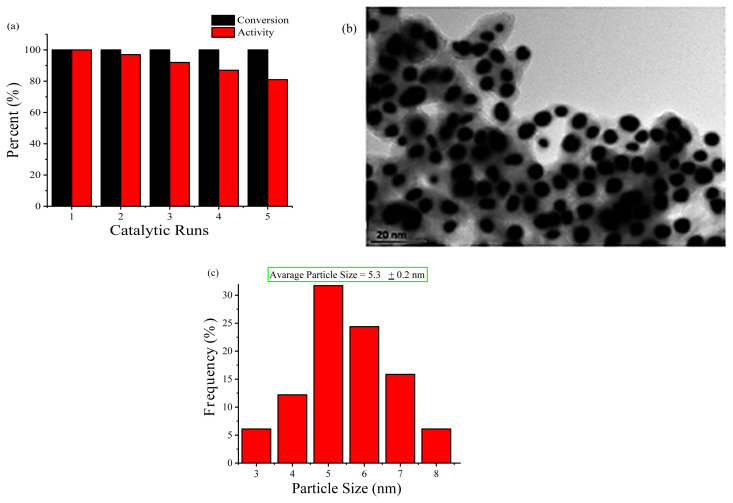
**(a)** The percentage of initial catalytic activity and conversion versus catalytic runs, **(b)** TEM image, and **(c)** particle size histogram taken after the 5th use for the Cu(0) NCats catalyzed green dehydrogenation of DMAB at nRT.

Reusability and isolability are two crucial criteria for heterogeneous catalysts. Therefore, the reusability and isolability of Cu(0) NCats were tested for the green dehydrogenation of DMAB at nRT. When dehydrogenation of DMAB catalyzed by Cu(0) NCats in the first run was ceased, black powder of Cu(0) NCats was isolated and then washed with 3×20 mL ethanol. The washed Cu(0) NCats was dried in vacuo and stored under inert atmosphere, and this process was repeated 5 times. It was found that the initial activity and conversion at the 5th run of Cu(0) NCats in the dehydrogenation of DMAB were 81% and >99%, respectively (Figure 11a). Also, the dihydrogen release achieved to 1.0 equiv. in the whole catalytic runs. The catalytic activity of Cu(0) NCats decreased by 19% in the 5th run. The increasing size of Cu(0) NCats because of the decrease in the number of atoms on the active surface and agglomeration of the metal can be the reason. TEM image (Figure 11b) and particle size histogram (Figure 11c) of Cu(0) NCats taken after the 5th use confirmed this condition, with the average size increasing from 2.9 ± 0.2 nm to 5.3±0.2 nm. Besides, poor mixing in the green environment might also have contributed negatively to the size growth of the metal. From these results, it was clearly understood that Cu(0) NCats exhibited quite high reusability and isolability in the green dehydrogenation of DMAB.

## 4. Conclusions

Consequently, in this work, an active, cheap, and readily available metal such as copper was used as a catalyst for the green dehydrogenation of DMAB at nRT. It was found that the green dehydrogenation of DMAB catalyzed by Cu(0) NCats exhibited very high catalytic activity, although an induction time (~45 min) was observed during the in situ formation of Cu(0) NCats after Cu(acac)_2_ reduction. Furthermore, the experiment of catalytic lifetime indicated that Cu(0) NCats was the long-lived catalyst with TON and TOF_app_ values of 456.5 over 50 h and 47.7 h^−1^, respectively, before deactivation in the green dehydrogenation of DMAB at nRT. As understood from the reusability and isolability studies, Cu(0) NCats was easily distributed in hexane, and its activity in the green dehydrogenation of DMAB did not change even after the 5th use. Also, its initial catalytic activity was retained by 81% even at the 5th run, while the full transformation of Me_2_NHBH_3_ to [Me_2_NBH]_2_ plus 1.0 equiv of dihydrogen was completed at nRT. On that account, this Cu(0) NCats having highly reusability and isolability is a good example for the green dehydrogenation of DMAB. The novel and remarkable contribution of this study to the literature is that no stabilizers, solvents, or supporters were used for the green dehydrogenation of DMAB in nRT. Moreover, the characterization of heterogeneous Cu(0) NCats has been reportedto be very economical with this new method using advanced spectroscopic techniques. It was observed that Cu(0) NCats with particle size of 2.9±0.2 nm exhibited rather high activity in green dehydrogenation of DMAB. Kinetic studies on the first and second sets showed that the green dehydrogenation of DMAB catalyzed by Cu(0) NCats is first-order with respect to catalyst amount, while it is about half-order with respect to DMAB amount. Also, the kinetic study on the 3rd set reveals that examination of temperature change is of great importance for calculation of the activation energy for dihydrogen release rate in the green dehydrogenation of DMAB catalyzed by Cu(0) NCats. As can be clearly seen in Table, the activation energy for the green dehydrogenation of DMAB catalyzed by Cu(0) NCats (E_a_= 16.6 ± 2 kJ mol^−1^) in the green medium at nRT is much lower than the one achieved by using both Ni(0) nanoparticles (E_a_= 42 ± 2 kJ mol^−1^) at 25.0 ± 0.1°C [26] and Ru NCs (E_a_= 18 ± 2, 45 ± 2, 25 ± 2 kJ mol^−1^ for Ru@PVP, Ru@PS-co-MA and Ru@Al_2_O_3_ NCs, respectively) at 35.0 ± 0.1°C [27]. The orders of activation energies for the above catalysts obtained for the similar reactions are lined up as Cu(0) nanocatalysts < Ru@PVP NCs ≈ Ru@Al_2_O_3_ nanoclusters < Ni(0) nanoparticles < Ru@PS-co-MA nanoclusters.

The very economical preparation of Cu(0) NCats in solvent-free media shows that it is an environmentally friendly and atom-economical catalyst in hydrogen production. It can be clearly stated that this method will open a new path for the design of transition metal catalysts with good dispersibility and high stability in the future and provide alternative catalysts.

## Figures and Tables

**Table:** Comparison of catalytic activity, particle size, and reusability catalysts reported plus activation energy of the catalytic dehydrocoupling of DMAB.

(Pre)catalyst	TOF (h^−1^)	Ea (kJmol^−1^)	Particle size (nm)	Reusability (Cycle)	Ref.
Dimethylammonium Hexanoate Stabilized Rh(0) NCs	60	34	1.9	aND	[14]
OAm stabilized Ru(0) NPs	137	29	1.8	75% (5th)	[21]
Pd NPs@Cu_3_(btc)_2_	75	173.5	4.3	80% (5th)	[45]
Ru NPs/ZIF-8	0.98	42.5	1.9	85% (5th)	[45]
*mer*-[Ru(N_2_Me_4_)_3_(acac)H]	0.3	85	aND	aND	[45]
bNi NPs	c21	42	1.9	63% (5th)	[26]
trans-[Ru(acac)_2_(OAm)_2_]	77.8	58	aND	aND	[45]
Pt(0)/OA@CNT	44.36	42.91	3.2	>80% (4th)	[45]
Pt(0)/DOA@CNT	57.32	40.23	3.4	>80% (4th)	[34]
Ru(0)/APTS	55	61	1.7	70% (6th)	[16]
Copper(0) NPs	71	88	aND	>54% (3th)	[25]
OAm stabilized copper(0) NPs	158	19	3.5	74% (5th)	[25]
bRu@PVP NCs	d56	18	12.9	87% (5th)	[27]
bRu@PS-co-MA NCs	d29	45	24.9	63% (5th)	[27]
bRu@Al_2_O_3_ NCs	d51	25	11.9	80% (5th)	[27]
b **Cu(0) NCats**	e **47.7**	**16.6**	**2.9**	**81%(5th)**	**This study**

a ND: not demonstrated.

b These nanoclusters were synthesized during green dehydrocoupling of DMAB.

c This TOF value was obtained within 4 h.

d These TOF values were obtained within 2 h.

e This TOF value was obtained after induction period within 50 h.
